# Laboratory-Based Resources for COVID-19 Diagnostics: Traditional Tools and Novel Technologies. A Perspective of Personalized Medicine

**DOI:** 10.3390/jpm11010042

**Published:** 2021-01-13

**Authors:** Boris G. Andryukov, Natalya N. Besednova, Tatyana A. Kuznetsova, Ludmila N. Fedyanina

**Affiliations:** 1 G.P. Somov Institute of Epidemiology and Microbiology, Russian Federal Service for Surveillance on Consumer Rights Protection and Human Wellbeing, 690087 Vladivostok, Russia; besednoff_lev@mail.ru (N.N.B.); takuznets@mail.ru (T.A.K.); 2 School of Biomedicine, Far Eastern Federal University (FEFU), 690091 Vladivostok, Russia; fedyanina.ln@dvfu.ru

**Keywords:** COVID-19, SARS-CoV-2, reverse transcription polymerase chain reaction (RT-PCR), enzyme-linked immunosorbent assay (ELISA), loop mediated isothermal amplification (LAMP), lateral flow immunoassay (LFIA), promising biomarkers, personalized medicine

## Abstract

The coronavirus infection 2019 (COVID-19) pandemic, caused by the highly contagious SARS-CoV-2 virus, has provoked a global healthcare and economic crisis. The control over the spread of the disease requires an efficient and scalable laboratory-based strategy for testing the population based on multiple platforms to provide rapid and accurate diagnosis. With the onset of the pandemic, the reverse transcription polymerase chain reaction (RT-PCR) method has become a standard diagnostic tool, which has received wide clinical use. In large-scale and repeated examinations, these tests can identify infected patients with COVID-19, with their accuracy, however, dependent on many factors, while the entire process takes up to 6–8 h. Here we also describe a number of serological systems for detecting antibodies against SARS-CoV-2. These are used to assess the level of population immunity in various categories of people, as well as for retrospective diagnosis of asymptomatic and mild COVID-19 in patients. However, the widespread use of traditional diagnostic tools in the context of the rapid spread of COVID-19 is hampered by a number of limitations. Therefore, the sharp increase in the number of patients with COVID-19 necessitates creation of new rapid, inexpensive, sensitive, and specific tests. In this regard, we focus on new laboratory technologies such as loop mediated isothermal amplification (LAMP) and lateral flow immunoassay (LFIA), which have proven to work well in the COVID-19 diagnostics and can become a worthy alternative to traditional laboratory-based diagnostics resources. To cope with the COVID-19 pandemic, the healthcare system requires a combination of various types of laboratory diagnostic testing techniques, whodse sensitivity and specificity increases with the progress in the SARS-CoV-2 research. The testing strategy should be designed in such a way to provide, depending on the timing of examination and the severity of the infection in patients, large-scale and repeated examinations based on the principle: screening–monitoring–control. The search and development of new methods for rapid diagnostics of COVID-19 in laboratory, based on new analytical platforms, is still a highly important and urgent healthcare issue. In the final part of the review, special emphasis is made on the relevance of the concept of personalized medicine to combat the COVID-19 pandemic in the light of the recent studies carried out to identify the causes of variation in individual susceptibility to SARS-CoV-2 and increase the efficiency and cost-effectiveness of treatment.

## 1. Introduction

In late 2019, the first cases of a new viral infection referred to as COVID-19 were recorded in China. Its causative agent is a previously unknown and highly contagious type of coronavirus, SARS-CoV-2 [1,2]. The subsequent global-wide spread of the infection became a disastrous pandemic and provoked a severe crisis in the global healthcare system and economy [1]. This virus is easily transmitted between people via airborne droplets and, therefore, quickly distributed in densely populated areas. According to the European Center for Disease Prevention and Control, the number of COVID-19 cases around the world in November 2020 was over 62 million with more than 1.4 million deaths [2,3,4].

The similarity of the clinical manifestations of the infection to the symptoms of other acute respiratory viral infections, the probability of the asymptomatic form of the disease, as well as the relatively high contagiousness, complicate the epidemiological monitoring of the virus distribution. In this regard, the platforms created for effective COVID-19 diagnostics have become especially relevant, as they provide timely detection and treatment of diseased patients and monitoring of the epidemiological situation, using the experience of control of other recent virus epidemics [1,3,4].

The emergence of three new types of coronaviruses pathogenic for humans in the 21st century raises serious concerns. These are RNA-containing members of the family Coronaviridae, including MERS-CoV, the previously described causative agent of the Middle East respiratory syndrome (MERS, Jordan, 2012), and SARS-CoV-1, the causative agent of the acute respiratory syndrome (SARS, China 2002) [5,6,7]. In addition, certain strains of α- and β-coronaviruses (HCoV-NL63, HCoV-OC43, HCoV-229E, and HCoV-HKU1) can also cause respiratory diseases and intestinal or neurological disorders in humans [8].

A genome-wide sequencing and phylogenetic analysis showed that the causative agent of COVID-19 is a β-coronavirus of the same subgenus as the SARS-CoV that has a rounded shape with a diameter of 60 to 140 nm [5,9]. To successfully control a pandemic, in addition to studying the viral agent, it is necessary to identify the main mechanisms of infection and determine the key strategies for diagnosing the infection.

**Figure 1:**
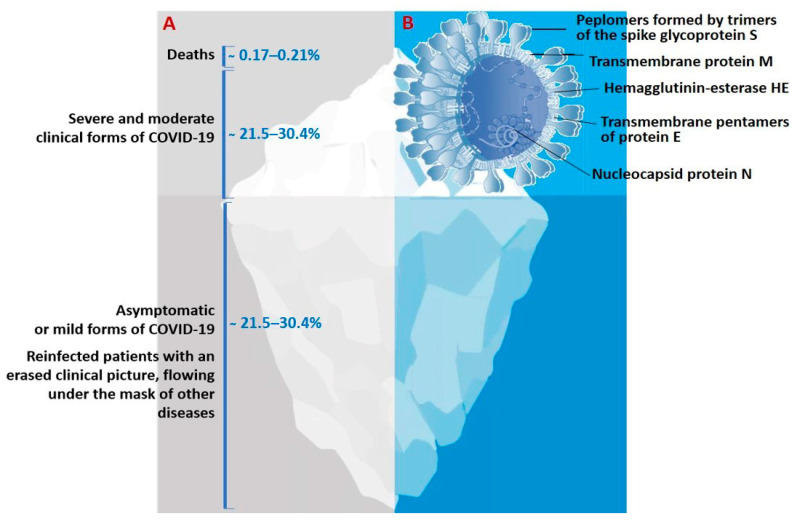
(**A**)The actual picture of the spread of COVID-19, resembling an iceberg and (**B**) some of the SARS-CoV-2 structural proteins of biotechnological and pharmacological interest.

The genome of SARS-CoV-2 is a single-stranded positive-sense RNA of 29,903 nucleotides [10,11]. This genome encodes as many as 27 structural and non-structural proteins that provide transcription and replication of the virus (genes *ORFlab* and *ORFla*), as well as its pathogenic effects. The viral proteome includes polyproteins, structural and non-structural proteins. Some of the structural proteins such as, primarily, the spike glycoprotein (S) exposed on the phospholipid membrane, as well as the envelope protein (E), membrane protein (M), and nucleocapsid (N), are of particular biotechnological, pharmacological, and biomedical interest [6,7,9,10,11,12] (Figure 1B).

The results of a molecular phylogenetic analysis showed that the genomes of SARS-CoV-2 and SARS-CoV, are related with an approximately 80% similarity. In particular, they share the largest of the structural proteins, glycoprotein S, which protrudes from the surface of mature virions. The S-protein plays key roles in virus attachment, fusion, and entry into human cells. For this purpose, the virus uses receptor-binding domain (RBD), which mediates binding to angiotensin converting enzyme 2 (ACE2) [11,13,14,15]. The highly immunogenic receptor-binding domain (RBD) of this protein is the main target for the neutralizing activity of antibodies and serves as a basis for the development of vaccines [16,17,18].

## 2. Laboratory Testing as a Basis for the Diagnosis, Treatment, and Monitoring of COVID-19

One of the most important issues in the strategy to control the new infection has been the necessity of mass laboratory-based screening of populations exposed to high risk of infection. The timely and high-quality laboratory-based diagnostics of patients infected by SARS-CoV-2 has become the top priority in eliminating the pandemic and taking quarantine measures [1,4,5,16]. Under these conditions, the creation of fast, effective, and inexpensive diagnostic tools is a necessary part of the fight against the new infection. When diagnosing COVID-19, the major challenge that the healthcare system faces is to identify the role and place of various diagnostic platforms for screening, diagnosing, and monitoring new coronavirus infections [8,9,19].

The results of the SARS-CoV-2 genome sequencing have become a basis for the development of vaccines and test systems to provide diagnosis and epidemic monitoring of the infection [19]. However, with lack of experience in eliminating the COVID-19 pandemic, the healthcare system now faces new issues and problems such as timeliness, frequency, and choice of testing tools, as well as identification of the place and role of their results in decision-making. These questions can be answered through solving the issues of availability of certain types of laboratory tests, timeliness of testing and their informativeness, and also the clinical, epidemiological and economic feasibility of their use in the rapidly changing and unprecedented pattern of the spread of the pandemic in recent history [15,20,21,22].

To date, there are substantial differences in the choice of optimal diagnostic tools and effective methods for testing patients with COVID-19, their contacts, asymptomatic vectors of the virus, medical specialists and other representatives of emergency medical services [1,2,19,22]. After nearly a year of fighting COVID-19, healthcare efforts are still measured in terms of number of tests performed [19,22].

The dynamics and sensitivity of laboratory tests for this category of patient remains unstudied, and they can become a source of infection for others [23]. This suggests that laboratory-based diagnostic strategies aimed at patients with symptoms are not sufficient to prevent the spread of the virus [20,21,22].

Negative polymerase chain reaction (PCR) tests and the detection of presence of specific antibodies are considered the criteria signifying a recovered patient, without considering the consequences on their health and quality of life. Nevertheless, levels of antibodies in those who have recovered are not further investigated, and the intensity of immunity and the possibility of re-infection by COVID-19 also remain unstudied [17,21]. This is probably due to the lack of understanding of the immune signaling pathways triggered by SARS-CoV-2, as well as the general immunopathology of this infection [2,3,22].

Consequently, a rapid, complete, and most accurate assessment of the spread of the virus requires laboratory-based tests for total screening of the population, which will allow rapid identification and isolation of infected patients. In addition, there is a necessity for a long-term strategy for preventing recurrent outbreaks of infection, which would imply repeated and regular mass testing of the immune response in the population to determine the effectiveness of vaccination [6,9,21,24].

The situation regarding the diagnostics of COVID-19 is further complicated by the lack of awareness in society, the mass media, among medical officials and some biomedical specialists concerning the differences between the existing types of diagnostic tests for this infection. Therefore, it is not surprising that neither a unified methodology with clear goals and objectives, nor an agreed interpretation of the results obtained yet exists [2,3,25].

Obviously, one of the major issues in the development of a testing strategy during the COVID-19 pandemic is associated with the existing types of diagnostic tool and their fundamental difference, clinical practicability, and uselessness for certain categories of patients at different stages of the disease. Other widely discussed issues are timing of testing, frequency, and correct interpretation of results obtained [2,3,5,7].

## 3. Laboratory-Based Tests to Diagnose COVID-19

Data obtained from routine laboratory examinations are non-specific (leukopenia, lymphopenia, mild thrombocytopenia, increased levels of acute phase proteins, decreased partial pressure of oxygen in the blood, and, in severe cases, identification of markers of cytokine storm in the form of increased levels of cytokines IL2, IL4, IL6, IL7, IL10, and TNF-α). These tests are helpful in treatment of patients diagnosed as COVID-19 positive [12,14,17,18].

All currently existing types of special laboratory tests for diagnosing COVID-19 can be divided into two categories: those that directly detect the virus (its genome or antigens) and those that detect the human immune response to its presence (antibodies IgM, IgA, and IgG).

Laboratory-based tests for COVID-19 are used for a variety of purposes. A diagnostic examination is carried out for patients with clinical symptoms (complaints) in order to confirm the diagnosis. A screening study is carried out for people who feel healthy in order to identify disease among them (including the asymptomatic form of infection). At last, monitoring is carried out for patients undergoing treatment in order to assess the effectiveness or dynamics of the latter [19,21,24].

With the lack of specific symptoms and lack of proven effectiveness of etiotropic treatment and vaccination methods, the results of special laboratory diagnostics are the only source of data to confirm the presence and provide monitoring of the progress of COVID-19 [3,23,24,26].

The main analytical characteristics of laboratory-based tests are their sensitivity (which is evaluated as the probability of positive result in a patient with the disease) and specificity (negative test results in a healthy person). In addition, the effectiveness of tests is evaluated by their predictive value: the post-test probability of the disease in persons with a positive test result and its absence in persons with a negative test result.

Most test system manufacturers report high analytical performance (90–100%) in cases in which their test systems are used under ideal conditions. However, in an actual situation, the diagnostic efficiency of a test depends on a number of factors (such as the clinical form of COVID-19, the duration of the disease, the quality of collection and the type of biomaterial, the conditions of its storage, transportation, etc.).

In an ideal (hypothetical) case, when using a test that detects SARS-CoV-2 and has 100% sensitivity and specificity, it would be possible to survey the entire world’s population. Depending on the results obtained, all infected patients can be sorted out and divided into the following categories: asymptomatic carriage and, depending on the clinical manifestation, mild, moderate and severe COVID-19. Accordingly, all patients with positive tests, depending on the clinical signs, are isolated either for quarantine, or home treatment, or treatment at a medical unit.

Alternatively, in another hypothetical case, the entire population is screened for the presence of IgG antibodies against SARS-CoV-2 using another test that has a 100% sensitivity and specificity to identify patients who were previously infected but had the asymptomatic form or were immune to the virus. These categories of the population, with their antibody level regularly monitored, could be recruited as volunteer to provide social or medical assistance to diseased people.

The actual pattern of distribution of COVID-19 resembles an iceberg, where the categories of seriously ill and hospitalized patients are in the smaller, above-water, part, and those who die from infection are at the very top (Figure 1). The largest proportion of the underwater part of the iceberg is represented by patients who have had an infection in an asymptomatic form of the disease which, depending on gender and age, account for more than 78%, or the mild form, without specific clinical manifestation, or with symptoms of acute respiratory or other flu-like infections [4,7,19,22,27] (Figure 1A). In this case, asymptomatic patients bear the same viral load for the same period of time as those with the pronounced form of infection and are considered as the main source of infection spread [3,4,7,19,22].

The cases considered above, being all hypothetical, help determine the position and assess the diagnostic value of the available tests, as well as the practicability of their use, especially with the lack of required therapeutic agents or vaccines.

## 4. Molecular Technologies for Identification of Nucleic Acids

When considering the range of diagnostic platforms, the techniques of viral cultivation and isolation of coronavirus or its antigens should be excluded from the first group of laboratory-based tests (that directly detect the virus). These studies are carried out in specialized virological laboratories through scientific research.

Detection of SARS-CoV-2 is an important diagnostic step in monitoring the incidence rate and managing the epidemiological process during the COVID-19 pandemic. The evolution of molecular/genetic methods for laboratory-based diagnostics is associated with the development of analytical technologies to identify nucleic acids, which have become rapid and reliable tools for the identification of RNA viruses [11,15,28,29,30].

The polymerase chain reaction (PCR) method is aimed at detecting deoxyribonucleic acid (DNA), one of two types of nucleic acids that provide storage of hereditary information in the genetic code that all living organisms have. Nowadays, PCR is a widely used diagnostic method for detecting a wide range of pathogenic microorganisms and is considered a “gold standard” for testing with high sensitivity and specificity [10,29,31,32,33]. For more than 30 years of application, this fundamentally simple technology has become one of the most common in biomedical practice for diagnosing infectious and hereditary diseases, as well as in molecular biology for scientific genetic engineering research [11,30,34,35,36].

The genome of SARS-CoV-2 consists of another type of nucleic acid, ribonucleic acid (RNA), which is similar in chemical composition to DNA, but has a number of fundamental differences. DNA stores hereditary information, and RNA transfer it; furthermore, DNA is a double-stranded molecule and is located in the nucleus, while RNA is single-stranded, located in cytoplasm, and has a lower molecular weight [32,36,37,38].

These differences explain the fact that the standard catalytic enzyme Taq polymerase, used in classical PCR technology to amplify (multiplex) certain DNA fragments in biomaterial, very inefficiently replicates RNA. Therefore, a different type of tests, referred to as reverse transcription PCR (RT-PCR), is used to detect RNA viruses, including SARS-CoV-2, due to its advantages of providing a specific and sensitive analysis for early diagnosis of viral infections [26,27,29,36].

In accordance with the recommendations of the US Food and Drug Administration (FDA) and the Centers for Disease Control and Prevention (CDC), when designing PCR test systems, RNA regions specific to SARS-CoV-2 (nucleocapsid N, genes E and ORF1ab, multimeric protein nsp12, RNA-dependent RNA polymerase, RdRp) are used [23,32,39].

Unlike traditional PCR applied to amplify target DNA sequences, RT-PCR is a technique for amplifying a specific RNA fragment into complementary DNA using the reverse transcriptase enzyme. RT-PCR is carried out in two stages: in the first stage, the RNA molecule is converted using a reverse transcription (RT) reaction, catalyzed by the enzyme reverse transcriptase (also known as reverse transcriptase or RNA-dependent DNA polymerase) into complementary DNA (cDNA), and then (second stage) the DNA molecule is amplified using Taq polymerase and the classical PCR scheme [11,31,33,40].

Depending on the number of samples and the organization of laboratory diagnostic process, the testing time ranges from 3–4 h to 6–8 h or more. For diagnostic purposes, it is most convenient to carry out these two stages in the same test tube; for scientific research, RT-PCR and PCR are carried out in separate tubes [34,37,39].

The RT-PCR method, which showed successful results in testing the seasonal influenza virus, has been approved by the World Health Organization (WHO) as a diagnostic standard for diagnostics and detection of SARS-CoV-2 [19,35,36,37]. Testing is carried out in a screening mode among asymptomatic patients with suspected contacts with SARS-CoV-2 carriers, for early detection or assessment of trends in the progress of infection [40,41,42,43].

In addition to the qualitative determination of RNA in viral infections, a technology combined with qPCR to quantify the expressed gene is also often used. This combined option, referred to as real-time quantitative RT-PCR (qRT-PCR or RT-qPCR), is the most informative and accurate modification of the “classic” PCR for detection and quantification of RNA level [33,35,38]. This method is quantitative (or semi-quantitative)—during the PCR process, the sample is constantly monitored by a sensor that records fluorescent signals at each cycle of the reaction. The resulting amplification curves are used in further analysis—they can be used to estimate the intensity of expression of certain genes and viral load [35,37,39].

RNA of the virus should be extracted after complete lysis of the biomaterial and purification from proteins, fats, carbohydrates, salts and cellular debris, cleavage by protease and elution of nucleic acids into solution. For virus RNA extraction, it is preferable to use filter column or magnetic bead methods that are of high purity [35,36,44,45,46].

There are a number of points to consider when interpreting the results of real-time PCR. One of the key values of real-time PCR is the cycle threshold (Ct), which occurs when the amplification curve crosses the instrument’s threshold line. The higher the value of the threshold cycle (Ct = 30–35), the less mRNA is in the sample, since it takes more time to amplify the number of mRNA copies required for intense fluorescence. If the Ct value is small (10–15), this indicates that the gene is expressed very actively. Usually, the internal control sample has a lower Ct value than the genes under study [35,36,39,44].

However, real-time PCR technology can quantify the viral load of COVID-19, which correlates with infectivity, disease phenotype, disease severity and mortality. For example, E. Pujadas et al. [45] prospectively quantitatively evaluated positive samples for SARS-CoV-2 by real-time RT-PCR. They calculated the viral load log_10_ in hospitalized patients, which averaged 5.2 copies/mL (surviving patients, n = 807) and 6.4 copies/mL in those who patients who died (n = 338). The authors concluded that quantifying viral load would assist clinicians in risk stratification and treatment choices [45].

The importance of monitoring viral load in patients with COVID-19 is of key importance for risk assessment, epidemiological control, and choice of therapy. To et al. [46] presented the results of a cohort study of the dynamic profile of viral load from samples of the posterior oropharynx in patients with severe COVID-19 (60–95 years, n = 23). Salivary viral load was highest during the first week after symptom onset and subsequently declined with time. Older age was correlated with higher viral load. The results indicate that the virus can be detected in small amounts of posterior oropharyngeal saliva despite clinical recovery (within 22–25 days) [46].

RT-PCR is one of the many varieties of traditional PCR techniques and is often confused with quantitative PCR (Q-PCR). The key difference between these two techniques is that, unlike RT-PCR applied to detect gene expression by generating cDNA transcripts from messenger RNA, Q-PCR is used for real-time quantification of PCR products using fluorescent intercalating dyes, or labeled probes [11,15,37,39].

Thus, the technology of PCR diagnostics has been improving for decades, with many trained specialists and the necessary equipment available, and the high sensitivity and specificity of this technology give reason to trust the results obtained. However, as often happens in clinical laboratory diagnostics, the importance of the preanalytical stage is overlooked, including collection and transportation of biomaterial, which, as a rule, is performed by untrained personnel or even by patients themselves (self-sampling, self-swab tests). This reduces confidence in results, especially if one uses screening assay [26,27,31,38,40,42].

The preanalytical stage in PCR testing is the key stage before performing tests for viral RNA. The proper timing of sampling, correct sampling, and optimal sample types are crucial factors for obtaining a correct result. CDC issued guidelines for collection, processing and clinical specimens for COVID-19, which outlined the basic requirements for the pre-analytical stage, types of samples for analysis, and collection of upper and lower respiratory specimens. These recommendations were recently (29 December 2020) updated [37].

For case screening for COVID-19 the CDC recommends the use of nasopharyngeal and oropharyngeal flocking swabs for SARS-CoV-2 molecular testing. Sampling with cotton swabs can wash out epithelial cells and reduce nucleic acid extraction. These samples are easier to collect, especially in resource-limited conditions [20,21,29,38,45]. It is extremely important to properly collect material in order to minimize the false-negative rate among COVID-19 positive patients. Samples should be obtained using flocking swabs and delivered to a laboratory as soon as possible after being collected.

However, posterior oropharyngeal saliva is increasingly recognized as a valid respiratory specimen for SARS-CoV-2 diagnosis. For example, Hung et al. [35] showed that using saliva from the back of the oropharynx collected early in the morning (immediately after waking up, before brushing teeth, rinsing the mouth and eating) to diagnose SARS-CoV-2 and the sensitivity of testing approached 91.7% compared to nasopharyngeal samples [35].

Samples should be obtained using flocking swabs and delivered to a laboratory as soon as possible after being collected. For patients with pneumonia, sputum and bronchoalveolar lavage (BAL) are additional materials for examination. To prevent RNA degradation, after being taken, swabs are immersed in a transport medium (lysis buffer or sterile saline). Samples should be stored at 2–8 °C for up to 72 h [11,35,36]. If longer storage is required, samples are frozen to −70 °C and below [35,37,38,39].

After collection, the smears are placed in a liquid medium to release viral RNA into it. Drying of the swab will affect subsequent extraction of the nucleic acid [11,15,20,29].

In each of the above-listed materials, the probability of detecting coronavirus varies and also may differ between patients; therefore, a negative test result does not rule out the probability of infection. It should rather be considered a screening test than a purely diagnostic test. As practical results of the use of PCR diagnostics show, the frequency of false-negative results can reach 40% [24,32,40]. In some cases, results of PCR diagnostics may be unconvincing despite the evident clinical symptoms, X-ray images, and epidemiological data.

The main explanation may be the uneven distribution of the virus in the respiratory system, as well as (inevitable in the presence of a huge flow of biological samples) the disregard of the standard sampling rules [35,36,39].

When performing the test, it should be taken into account that some components in the sample can reduce or completely inhibit the activity of the catalytic enzymes involved (transcriptase and Taq polymerase), which, accordingly, will partially or completely block the amplification of DNA molecules, reduce the quality of diagnostics, and become one of the factors responsible for false-negative PCR results [39,40]. A sign of PCR inhibition is the simultaneous absence of amplification of the internal control and the specific product. The following substances are classified as PCR inhibitors: hemoglobin contained in blood impurities, isopropyl alcohol and methyl acetate [13,34,39]. For example, Taq polymerase is completely inactivated in the presence of even traces of blood in the sample (from 0.004%). Therefore, the quality of sampling and sample pre-processing is a key stage in PCR testing, affecting the diagnostic reliability and efficiency of the method [34,35,39,40].

Sample quality is a fundamental issue in laboratory diagnostics. Therefore, common to all diagnostic platforms is a new trend in quality, resulting in a more sophisticated mode, either a laboratory-developed or point of care commercial test [34,35,40].

The material taken for analysis should contain the maximum concentration of the target microorganisms and should be free of undesirable impurities that inhibit PCR. Unfortunately, the design of the qPCR analysis does not include quality control standards for analysis of samples of the Sample Adequacy Control (SAC) type, represented by a single-copy of the human genome, which ensures the optimal quantity, quality and conformity of samples delivered for research [39]. In addition, the use of such control gives confidence in the reliability of a negative result, without which the effectiveness of screening is jeopardized [39,40]. This type of in-laboratory control (in a ‘competitive’ or ‘non-competitive’ format), along with other control tools used in molecular testing, is critical to address a variety of analytical sample-related problems. This should become a routine sample quality control tool, especially relevant during the COVID-19 pandemic.

Over time, changes appear in the SARS-CoV-2 genome, there is a sequence mismatch between primers and probes, and, therefore, these sites can affect the sensitivity of molecular tests. In this connection, on the recommendation of WHO, the monitoring of mutations of the coronavirus is carried out [42]. Most mutations do not have a noticeable effect, but some, formed in the binding regions of the primers and probes of SARS-CoV-2, can reduce the sensitivity and accuracy of the used PCR test kits and lead to false negative results [22,34,43,45].

One way to reduce the risk of false negative test results due to the appearance of new SARS-CoV-2 mutations is to routinely examine all samples of the material using two different sets of primers (probes) that identify the nucleocapsid (N) gene and one of the additional genes (proteins E, S or specific RNA-dependent RNA polymerase) as targets [43,44]. In addition, a number of tools are available to monitor SARS-CoV-2 mutations, including a database search tool such as GenBank, NGDC Genome Warehouse and CoV-GLUE [42,44,45].

Thus, Khailany et al. analyzed 95 genomic signatures of SARS-CoV-2 [44] deposited in GenBank and the NGDC Genome Warehouse. The analysis revealed 116 mutations, of which the most common were associated with the ORF1ab and N genes (encoding the synthesis of structural protein N). The authors conclude that these mutations may affect the quality of diagnosis and the spread of SARS-CoV-2 [44].

Recently, a new phylogenetic cluster SARS-CoV-2 (B.1.1.7) was discovered in the UK, which is characterized by more contagiousness and more genetic changes, especially in the spike protein. Soon this cluster was found in other countries, which indicates its rapid spread [47].

Thus, the risk of false-negative, as well as false-positive, results is an important problem associated with the method of real-time RT-PCR that significantly affects the effectiveness of control over the spread of infection [42]. Wang et al. [39,43] report that many cases of COVID-19 with a typical clinical manifestation, and the corresponding result of a computed tomography (CT) scan, were not diagnosed by PCR testing [39,43].

To eliminate or minimize the likelihood of false-positives, it is recommended that a nuclease-free negative template control (NTC, No Template Control) be used with each cycle of SARS-CoV-2 RT-PCR testing. NTC test results should be negative, and positive results will indicate sample cross-contamination [12,35,37,47].

Thus, real-time RT-PCR testing of SARS-CoV-2 is the method of choice in the diagnosis of COVID-19. In mass and repeated studies, these tests not only identify infected patients, but also diagnose COVID-19 in asymptomatic carriers and, therefore, more accurately determine infection rates in the population. However, RT-PCR results should be interpreted with caution, taking into account CT data and clinical symptoms of the disease, which facilitate early diagnosis and ensure timely treatment of patients. The efficiency of the method is greatly enhanced by using multiple type of samples collected from the upper and lower respiratory tract, and also by following standard procedures and laboratory practice.

Nucleic acid tests are complemented by serological methods.

## 5. Serological Methods for Detecting Antibodies and Determining Protective Immunity in SARS-CoV-2 Infected Patients

The second group of widely used methods consists of tests designed to detect infection-related antibodies of the IgA, IgM, and IgG classes, and also to determine protective immunity in various categories of population infected by SARS-CoV-2. It is recommended to collect venous blood for serological testing without anticoagulant, to avoid hemolysis, and to avoid bacterial contamination and the influence of fibrin. Antibody test results are especially important for detecting a previous infection in patients with the mild or asymptomatic form of disease [2,46,48,49].

Serological tests, in the most common format of the enzyme-linked immunosorbent assay (ELISA), are well known and have proven to be relatively simple, safe, sensitive, and specific diagnostic platforms for detecting antibodies in serum or plasma produced in response to SARS-CoV-2 [23,46,49,50,51].

When developing test systems for detecting antibodies to SARS-CoV-2, recombinant antigens are currently used, which are more often used as immunogenic structural proteins of coronavirus—nucleocapsid (H) and spike glycoprotein (S) [23,49,50].

The full research cycle takes at least 1.5–2 h and requires special laboratory equipment (automatic ELISA analyzer or (in simplified format) microplate spectrophotometer, thermostatic shaker and tablet washing device) [23,49,50,51,52,53].

**Figure 2:**
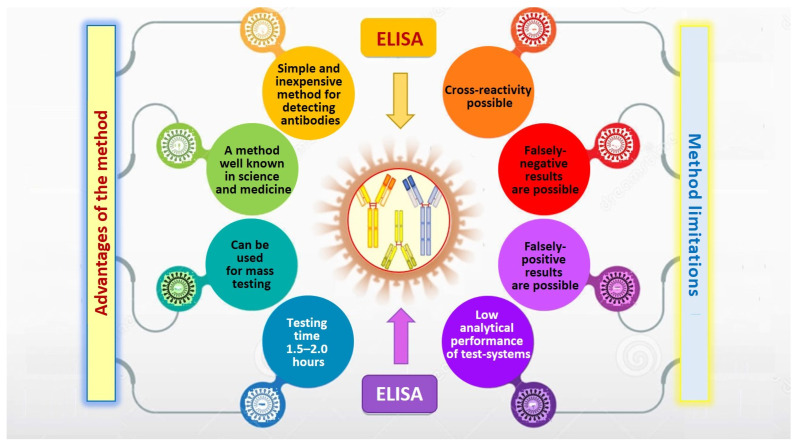
Advantages and limitations of the enzyme-linked immunosorbent assay (ELISA).

The main requirement for the test systems is no cross-reactivity with antibodies produced against other common coronaviruses that cause less serious respiratory diseases (HCoV-NL68, HCoV-OC43, and HCoV-HKU1). However, a potential cross-reactivity cannot be completely ruled out when conducting ELISA tests. False-positive results may occur in the case of infection caused by another type of β-coronavirus, if nucleocapsid N is used as an antigen in test systems [46,49,50,51,52]. According to manufacturers, most of the serological ELISA tests have a specificity of more than 99% and a sensitivity of 96% or more [54,55]. However, most authors, in order to avoid false-negative results, point to the need to study antibodies against several antigens and also to analyze samples collected within 20 days after infection or after the first symptoms are recorded [2,23,46,49] (Figure 2).

A recent comparative analysis of the dynamics of the appearance of the corresponding antibodies showed that, 14 days after the onset of symptoms, antibodies against protein H SARS-CoV-2 showed 100% sensitivity and 100% specificity, while antibodies against protein S were detected with a sensitivity of 91% and specificity 100% (IgM and IgG, n = 412, accuracy of 97.5%) [51].

The dynamics of the immune response against SARS-CoV-2 and, in particular, seroconversion (the production of antibodies and their detection in the patient’s blood serum) in cases of COVID-19, as well as the correlation of the level of specific antibodies with the viral load and their role in the elimination of the virus, are still under study [7,23,46].

Antibody detection is important for patients who present late with very low viral load on RT-PCR. In the previously mentioned study [46], it was found that the levels of antibodies in blood serum against nucleoprotein (NP) and the binding domain of the surface spike protein receptor (RBD) SARS-CoV-2 did not correlate with the severity of COVID-19.

In most patients, 14 days or longer after symptom onset, seropositivity rates were 94% for anti-NP IgG (n = 15), 88% for anti-NP IgM (n = 14), and 100% for anti-NP IgG. RBD IgG (n = 16) and 94% for anti-RBD IgM (n = 15). Anti-SARS-CoV-2-NP or SARS-CoV-2-RBD IgG levels correlated with the virus neutralization titer [46].

SARS-CoV-2 neutralization tests are critical to assess the effectiveness of the immune response. However, this test is logistically demanding, time-consuming, and requires level-3 containment facilities to safely work with live virus. Employees of the Vistar Institute and Inovio Pharmaceuticals have recently proposed two safe tests to detect virus-neutralizing antibodies. The first assay is surface plasmon resonance (SPR) based and can quantitate both antibody binding to the SARS-CoV-2 spike protein and blocking to the ACE2 receptor in a single experiment. The second assay is ELISA based and can measure competition and blocking of the ACE2 receptor to the SARS-CoV-2 spike protein with anti-spike antibodies. This type of rapid, surrogate neutralization diagnostic can be employed widely to help study SARS-CoV-2 infection and assess the efficacy of vaccines [53].

In addition, detection of the correlation between neutralizing antibodies, clinical course, prognosis, and temporal pattern of immunoglobulin production will be relevant in identifying specific therapeutic agents for the treatment of COVID-19 [54].

A strong positive correlation has been found between clinical severity and total antibody titer within two weeks after the symptoms appear. According to preliminary data [7,56,57,58], the current pandemic is characterized by a typical temporal pattern of production of immunoglobulin classes (Table 1).

Guo et al. [58] note that the mean time of appearance in blood of low-affinity IgM-specific antibodies is 5–7 days, while high-affinity IgG and IgA antibodies were detected within 14–20 days after the onset of symptoms in individuals with the RT-PCR-confirmed infection [58]. Furthermore, the protective properties of antibodies against reinfection of patients who have recovered from COVID-19 and the duration of persistence of protective immunity also require confirmation [20,23,53,55,56,57].

Studies on the dynamics of the emergence of antiviral antibodies in patients with other coronavirus infections during the SARS and MERS epidemics have shown that specific antibodies are produced in 80%–100% of patients within, on average, 14–20 days after diagnosis, which corresponds to 2–3 weeks after the onset of the clinical symptoms of the disease [9,12,26,49,50,51,52,53]. It has been convincingly demonstrated that seroconversion is as reliable a diagnostic indicator of coronavirus infection as detection of viral RNA [11,49,52,53,54].

Studies of the immune response of infected patients during the current pandemic show that the results of serological tests for antibodies against SARS-CoV-2 can be used to retrospectively identify patients who have had the asymptomatic and mild forms of the infection. In addition, serological tests allow monitoring of the progress of infection in hospitalized patients, as well as tracing contacts and providing epidemiological surveillance at the regional level to determine the actual scale of the pandemic and the mortality rate [23,46,49]. The appearance of specific antibodies in medical specialists, exposed to potentially dangerous conditions, through re-infection by COVID-19 could serve as a criterion for the admission to work [2,3,7,49]. Finally, the study of seroconversion in SARS-CoV-2 infected persons can be used to diagnose PCR-negative patients, including those hospitalized at a late stage of the disease, and to assess the sensitivity of molecular/genetic testing.

Thus, serological testing is carried out after the acute period of disease or exposure to the virus in order to assess the immune system response to the infection, its duration, and the response to vaccination. In a broad sense, testing for the presence of antibodies in a population is carried out to study the population immunity (effects of individual immunity in a population context) [59].

## 6. Search and Development of New Methods for Rapidly Diagnosing COVID-19

During the pandemic, having simple, rapid, and mass testing methods for detecting SARS-CoV-2 infection is critical in order to take effective anti-infection measures [6,13,52,53]. Mass detection based on standard diagnostic RT-PCR tests using throat and nasal flocking swab samples has proven to be very effective in combating the COVID-19 pandemic worldwide. However, the use of this testing method implies huge financial costs due to the purchase of the necessary expensive equipment, test systems, and construction of specialized laboratories. In addition, in the context of centralized laboratory-based diagnostics, the testing procedure takes several days [23,52,53,54].

With limited resources and equipment, the lack of opportunities to conduct routine laboratory diagnostics of COVID-19 is becoming a factor that limits the efficiency of efforts to control the spread of the virus. Therefore, the search for, and development of, diagnostic tools corresponding to the ASSURED concept (Accessible, Sensitive, Specific, User-friendly, Fast, REliable and no-Device) is an urgent objective [13,23,52,54].

One of the modern hi-tech trends in indication and identification of infectious pathogens is the development of point-of-care (POC) technologies in clinical laboratory diagnostics. The advantages of POC technologies, such as simplicity, rapid results, cost effectiveness, and no need for trained specialists and expensive laboratory equipment, reduce the cost of coronavirus detection and mediate their active application in diagnostics [52,53,54].

A number of molecular methods from the range of POC technologies have already been developed and are available for rapid diagnosis of patients with COVID-19. One of these methods is the detection of viral RNA based on the technology of Loop mediated isothermal amplification by reverse transcription (RT-LAMP).

***Loop mediated isothermal amplification (LAMP).*** Recently, the method of Loop mediated isothermal amplification with reverse transcription (LAMP and RT-LAMP) has become increasingly popular for screening and monitoring diagnostics of viral infections, similar to the classical PCR [23,28,60,61]. This diagnostic platform, introduced in 2000, has been recognized as a fast, simple, cost effective and highly efficient testing method [23,28]. It can be used for remote screening of biological samples outside of centralized laboratories, as well as for monitoring isolated populations [23]. LAMP and RT-LAMP technologies have worked well in the past for identifying various viruses, including SARS-CoV, MERS-CoV [62], and Ebola [63].

This one-step amplification method detects the presence of viral RNA in various bio-substrates with an accuracy comparable to that of RT-qPCR. The latter, being the gold standard for SARS-CoV-2 detection, nevertheless requires trained personnel, complex infrastructure and long analysis times, which limits its use [64,65,66]. During the COVID-19 pandemic, such new and cost-effective laboratory POC technology as RT-LAMP, being a simple screening tool in resource-limited settings, has become a worthy alternative to RT-PCR [64,66,67,68].

**Figure 3:**
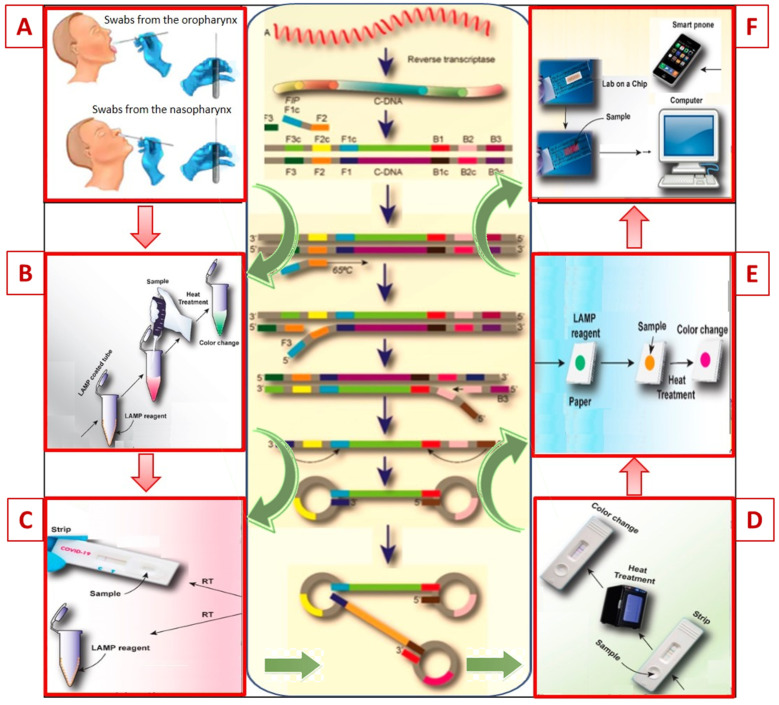
Schematic representation of the amplification sequence for loop mediated isothermal amplification by reverse transcription (RT-LAMP) and future prospects of this technology for the diagnosis of COVID-19. (**A**)—for diagnostic COVID-19, samples can be collected by a nasopharyngeal and oropharyngeal swab or sputum; (**B**,**C**)—the samples mixed with the LAMP reagents at 60–65 °C for 30 min to complete the amplification; (**D**,**E**)—visual assessment of the LAMP result using the calcein dye colorimetric reaction reduced the study time to less than 30 minutes; (**F**)—full automation of LAMP methods is possible with connection to an electronic device (computer or smartphone) for real-time transmission of the result.

Similar to the RT-PCR method, LAMP tests are very sensitive and reliable when used during the acute phase of infection, with results obtained within 20–60 min. A key step in the development of RT-LAMP test systems is the selection of targets of the virus and the use of the appropriate four primers targeting different conserved regions of the genome [64,65,68]. The primers bind to target complementary DNA (cDNA) sequences and form dumbbell shaped DNA. Then, in the stage of cyclic amplification, several copies of such DNA dumbbells are continuously produced. The products formed at the stage of cyclic amplification are used in the elongation phase for cyclic synthesis of DNA of various sizes (Figure 3).

Recently Lamb et al. [64] proposed a one-stage LAMP rapid test for the diagnosis of COVID-19 with an analysis time of 30 min. In the test kit, primers N1 and N15 were used, targeting the genes SARS-CoV-2 encoding the N nucleocapsid, as well as the spike glycoprotein S (*S17* and *O117*) and replicase (*ORF1ab*), respectively. A 100% specificity and high sensitivity of the test was confirmed on samples of serum, urine, saliva, swabs from the oropharynx and nasopharynx, using colorimetric and fluorescence detection [64].

In another study by Park et al. [65], it was shown that primers targeting the *Nsp3* gene, in combination with primers targeting the *N* and *S* genes, give significantly better results and the shortest cut-off time for cDNA production [65].

The technological advantages of LAMP, such as amplification at a constant temperature of 60–65 °C (which eliminates the need for a thermal cycler and allows the reaction to be carried out in a simple water bath) and faster results, while providing similar sensitivity and specificity, make it more suitable than RT-PCR for monitoring the COVID-19 pandemic [64,67,68].

In addition, the important advantages of this unique method are high efficiency and ease of implementation with the opportunity to identify the amplification product and evaluate the result visually by color change after adding one of the intercalating dyes [67,68]. Modern modifications of RT-LAMP technology allow obtaining a quantitative result in a real-time mode using smartphone cameras or scanners [60,61,68] (Table 2).

An important advantage of this one-step method is the opportunity of testing in a wide range of temperatures and pH values, which facilitates and reduces the time of the sample preparation stage [60,61,62,65,66,67].

An example of a successful clinical experience in creating an effective tool for diagnosing COVID-19 is the N1-STOP-LAMP test developed by Australian scientists, based on the RT-LAMP method with a set of primers for detecting the CDC N1 region of the SARS-CoV-2 nucleocapsid (N) gene. When tested on clinical specimens, previously tested by RT-qPCR with the E-gene, the new test system showed an 87% sensitivity and 100% specificity with an average time of detecting a positive result of 14 min [69].

A limitation of application of the LAMP testing technologies is the lack of sufficient experience of researchers in using the method under conditions of epidemic outbreaks and emergency situations, as well as in the clinical interpretation of the results. In addition, the designing of LAMP test kits is a more complex process, requiring multiple primer pairs, which slows down clinical use during epidemics [62,63,65]. The rapid development of this promising technology is facilitated by the appearance of new commercial LAMP test systems (RT-LAMP), including those approved for SARS-CoV-2 detection in a large number of samples, on the medical equipment market (Table 3).

The cost of LAMP per test is also considerably lower than other available molecular tests [60,61,62,66,67,68]. During a pandemic, significant research efforts are focused on optimizing and modifying existing RT-LAMP platforms for diagnosing COVID-19 in underdeveloped countries with limited resources and in the absence of centralized laboratories [61,66,67].

The promising future of LAMP technologies as a tool for diagnosing COVID-19 is evidenced by the latest developments that combine the potential of the method with the CRISPR-Cas12 molecular immunity system (13), of which collateral activity against RNA targets of viruses provides the basis for highly specific and sensitive detection of SARS-CoV-2 [72]. This points toward the potential revolutionary role and benefits of modified RT-LAMP over conventional PCR as a POC tool in the current COVID-19 pandemic and similar pandemics in the future.

Recently, more attention has been attracted by a new LAMP technology, based on ligation (Ligation-LAMP), where only two primers are needed and two dumbbell ligation oligos [67]. A unique design in this strategy is the use of high-fidelity ligase to construct a template that can be efficiently amplified utilizing LAMP reaction. This modification of LAMP uses template cDNA ligation of two hairpin probes, followed by LAMP-mediated detection of the ligated product. The method provides highly selective and sensitive quantitative detection of RNA in a dynamic range of 1 fM to 1 nM [67,68]. The developed Ligation-LAMP assay strategy presents promising prospects for the hypersensitive detection of SARS-CoV-2 mutations [68].

***Lateral flow immunoassay (LFIA) for the diagnosis of COVID-19.*** Another promising technology for the rapid diagnosis of coronavirus infections and, in particular, COVID-19 is lateral flow immunoassay (LFIA), which is also known as immunochromatographic analysis (ICA) [7,26,50,52]. This diagnostic technology based on immuno-serological testing has been widely known for over 60 years. Subsequently, this simple and cost-effective POC platform has been continuously developed, in particular, for the purpose of diagnosing bacterial and viral infections [52,73,74].

The special attention to LFIA technology has been influenced by the explosive growth of sensor diagnostic systems. Depending their technological capabilities and current diagnostic solutions, ICA platforms are considered as simplified formats of modern biosensors. The design of LFIA tests allows nucleic acids, aptamers, or antibodies to be placed in the test (bioreceptor) zone located on the sensor substrate. Thus, the existing formats of LFIA test systems make it possible not only to detect antigens in bio-substrates, but also to determine the presence of antibodies (as well as specific proteins and enzymes) in blood. In this sense, these technologies occupy an intermediate position between molecular/genetic and serological diagnostic tools [7,26,50]. The short detection time, simplicity and cost-effectiveness are the main advantages of LFIA testing. For example, unlike ELISA, the entire cycle of antibody testing using LFIA test systems takes from 10 to 20 min [7,52,73,74].

These rapid diagnostic tests have shown successful results in diagnosis of SARS, MERS and COVID-19 coronavirus infections in the most commonly used format for detecting the presence of specific antibodies (IgM and IgG) or viral antigens in patient bio-substrates [26,52,74,75,76]. Thus, LFIA tests make it possible not only to identify infected patients, but also to carry out retrospective diagnosis of infections in patients with asymptomatic or mild COVID-19 [7,74,75,76].

**Figure 4:**
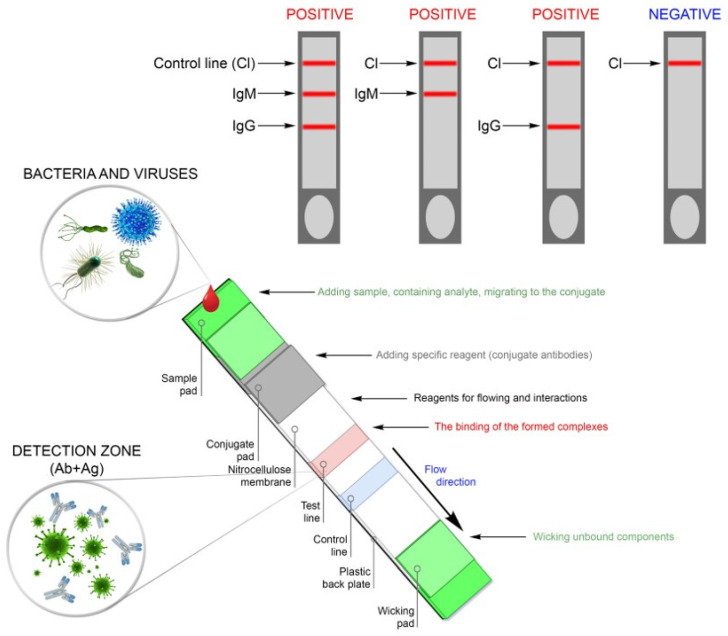
Schematic representation of the construction of the lateral flow immunoassay (LFIA) test system and interpretation of the results.

In the serological format for the detection of antibodies against SARS-CoV-2, LFIA test systems are manufactured in the form of kits to be handled by specialists in the clinical diagnostic laboratories of hospitals and outpatient clinics. The test requires a drop of patient’s blood, serum, or plasma. The sample is applied to a pad, then specific reagents (conjugated antibodies) and reagents to promote and interact with antigens and antibodies against SARS-CoV-2 present in the sample are sequentially added. The results are read visually by the appearance of color stripes in the control and test zones (Figure 4).

Wu et al., in order to study the dynamics of seroconversion in COVID-19, as well as to assess the sensitivity and specificity of four LFIA test systems, conducted a retrospective study of antibodies to SARS-CoV-2 in patients diagnosed by molecular testing (RT-PCR). It turned out that three weeks after the onset of symptoms of the disease, all tests revealed antibodies (IgM and IgG) in patients’ blood sera. Moreover, in patients with COVID-19 complicated by pneumonia, an earlier appearance of antibodies against SARS-CoV-2 was revealed [7].

In another study, Chen et al. [26] reported the development of a new LFIA test system based on lanthanide-modified polystyrene nanoparticles and a recombinant SARS-CoV-2 nucleocapsid protein placed on a nitrocellulose membrane to capture specific antibodies. The entire analysis process took 10 min [26].

Unlike enzyme immunoassay and molecular methods, the use of LFIA tests does not require special training of personnel. These tests can be used for screening studies in the field, at railway stations, airports, private clinics, unequipped diagnostic units, and in rural hospitals [73,75,76].

Although the use of individual LFIA test kits is more expensive than ELISA for diagnosing SARS, MERS, and COVID-19 infections, this has proven to be economically justified by the clinical benefits of this diagnostic platform [20,25,73,74].

In recent months, to control the COVID-19 pandemic, many LFIA test systems have been developed and proposed, which differ in their main analytical characteristics [74,75,76]. Some of the proposed diagnostic systems have passed the necessary expertise requirements and received permission for use in clinical practice (according to Johns Hopkins University) [77] (Table 4).

Recently, the well-known British company Avacta Group plc, a manufacturer of biotherapeutic drugs and reagents, announced its new development of a rapid test based on the LFIA technology with the aim of detecting SARS-CoV-2 antigen in patient saliva within a few minutes. This test has the potential to be used for frequent mass testing of the population to promptly identify infectious individuals [85].

The robust efficacy and high sensitivity of ICA in detecting SARS-CoV-2 specific antibodies indicates that these technologies can be a useful diagnostic tool in addition to molecular methods for diagnosing COVID-19 [86,87].

International experience of using serological tests based on LFIA to eliminate epidemic outbreaks of coronavirus infections has shown the importance and necessity of this diagnostic tool. Its most rational application is mass screening of the population from risk groups, as well as patients with an asymptomatic form of the disease. All positive results must be confirmed by quantitative molecular/genetic methods.

## 7. A Year of Fighting the COVID-19 Pandemic: Is It Already Time for Personalized Medicine?

The beginning of the XXI century was marked by the beginning of the post-genomic era and the emergence of new analytical approaches. Mono-marker diagnostics have been replaced by an innovative strategy aimed at obtaining and using complete profiles of all molecular determinants of biological systems [88,89,90]. The joint progress of new analytical platforms and biostatistics made it possible to create advanced omix-technologies covering the entire field of molecular cell biology, including genomics, proteomics, lipidomics, transcriptomics, and metabolomics [89,91]. Each of these technologies studies a different group of molecules, using their own tools to assess specific profiles in specific systems, tissues or cells, clinically using a scientific approach “not based on hypotheses” [88,90].

In recent decades, an increasing number of omix-technologies have become available for clinical diagnostic laboratories, where they are used for diagnosis, dynamics, prediction and assessment of the pathophysiological mechanisms of the disease [89,91,92]. New notions that have appeared recently—molecular and metabolic profiling, genomic screening—are now used in the diagnosis of pathology in patients with nonspecific clinical manifestations, when it is impossible to accurately determine the only biomarker mediating the disease [88,89,91].

A modern product of high-performance advanced omics-technologies is personalized laboratory medicine, which, unlike unified laboratory medicine, does not rely on a specific diagnostic marker, but is based on a change in the profile of many markers [88,89]. This approach allows an individual molecular (genomic, metabolomic, proteomics, transcriptomics, lipidomics, transcriptomics) portrait of a patient [88,90]. A personalized spectrum of omix-data mediates a predisposition to a specific pathology, the unique clinical course of the disease, prognosis, response to therapy, vaccination, etc., as well as providing timely and individual treatment and targeted prevention [89,91,92].

For example, the measurement and search for new biomarkers using “-omics” (genomics, proteomics, metabolomics) serves the timely identification cellular stress molecules that signal pathology. The study of molecular stress signals is a promising area for the search for new biomarkers, including viral activation [88,90].

The so-called stress signals, biologically active molecules that trigger a cascade of cellular reactions, testify to cellular ill-being during viral infection. Their appearance can serve as an individual stress marker of the influence of SARS-CoV-2 on the dynamics of the development of the infectious process at the molecular and cellular levels in individual patients with COVID-19 [90,92].

For example, Battagello et al. [93] summarized data on the significance of cellular stress signals during penetration and into the cell and replication of SARS-CoV-2 and concluded that the appearance of clinical symptoms in individual patients depended on the predominant lesion and invasion of the virus into cells, where ACE2 is considered the main receptor for the protein spike (S-protein), through which the virus can attach to the target cells [93].

It has been established that molecular markers of the penetration of SARS-CoV-2 into host cells can be increase in the activity of serine protease 2 and modulation of the activity of the renin-angiotensin system, which are closely related to ACE2 [92,94,95,96]. The same target is used to assess the effectiveness of a drug in model systems and the interaction of metabolic enzymes [97].

In clinical practice with infectious diseases, physicians frequently face the phenomenon of variation in individual susceptibility between patients with the same disease caused by an identical pathogen. This variation depends on a combination of a number of individual factors such as gender, age, the presence of comorbidities, and genetic polymorphism. These unique set of biological variables determines individual response to infection and underlies the concept of personalized medicine. With the progress of SARS-CoV-2 study, many new questions arise that remain unanswered. Perhaps they require a deeper understanding of the pathophysiological processes in COVID-19.

A number of issues related to the course, diagnosis and prevention of COVID-19 can be resolved within the framework of the new concept of personalized medicine. It is a known fact that a person has individual genetic determinants of susceptibility to certain infections, mediating both resistance and high susceptibility to disease. In the first months of the current pandemic, the heterogeneity of the clinical manifestations and of the severity of the course of the new coronavirus infection was revealed. It was soon found that older age and male sex were powerful factors associated with the incidence of SARS-CoV-2 infection and the more severe clinical picture. However, these risk factors did not fully explain the wide range of clinical manifestations and patterns of COVID-19, ranging from asymptomatic and mild COVID-19 to severe pneumonia, respiratory failure and death [92,94,95,97].

It was found that the most common cause of a weak response of the organism to the introduction of the virus is congenital genetic determinants or aging of the immune system (immuno-senescence) in elderly patients, which can contribute to the observed diversity of the severity of the disease and lead to dysfunction of antiviral immunity [95,97,98].

It is necessary to carefully examine the patient’s molecular profile with determination of the state of all links of his immunity, in order to timely identify genetic predisposition to infectious diseases before infection or in the early stages. This will make it possible to identify a risk group with a low or high probability of developing complications of a particular disease, predict an individual response to immunomodulatory therapy, and predict the likelihood of developing side effects to antiviral therapy [83,90,98,99,100].

For example, Zeberg et al. [98] identified those carrying the risk allele for SARS-CoV-2 infection at rs35044562 (“neanderthal haplotypes”), associated with severe disease and death in COVID-19 [98].

With the increased introduction of molecular diagnostic methods, the cost of separate study is decreasing and over time in infectious diseases hospitals it will become possible to use microchips with a panel for detecting genetic polymorphisms associated with hypersensitivity to commonly used antibacterial or antiviral drugs, which will prevent a large number of deaths [88,90,91,99].

In this regard, one of the goals of modern omix-technologies is to develop molecular profiles of bio-substrates, such as, e.g., the protein profile of pathological urine or the metabolic profile of selected body fluids [88,90,100].

A recent study by Liu et al. [101] evaluated the diagnostic role of proteomic analysis of urine samples in assessing the progression of COVID-19 infection, from mild to severe clinical forms, and recovery using mass spectrometry. The revealed protein spectra showed that, in patients with a severe course of infection, the content of proteins associated with complement activation and hypoxia was greatly increased, while the level of proteins associated with platelet degranulation, as well as glucose and lipid metabolism, were decreased. [101].

In particular, the authors drew attention to the dysregulation of metabolism and transport of lipids in patients with COVID-19, which confirms the results of studies conducted earlier [102] in which it was found that impaired cholesterol homeostasis negatively affects the prognosis of COVID-19 and the effectiveness of antiviral therapy [101,102]. Based on the results obtained, the authors concluded that the assessment of the urine proteomic profile could potentially be used to differentiate and predict the course of COVID-19. In addition, characterization of the protein spectrum can help in understanding the pathogenesis of the new coronavirus infection [102].

Another aspect associated with immuno-senescence is mediated by mitochondrial dysfunction. It is this self-autonomous subcellular organelle, in addition to its various intracellular functions, which directly regulates the activation of innate antiviral immunity [103,104]. It was found that studies of the energy-metabolic status in patients with coronavirus infection not only reflect one of the main pathogenetic strategies of SARS-CoV-2 associated with the colonization of the host’s mitochondria, but also assess the status of anti-viral immunity [87,88,89]. Therefore, an individual assessment of mitochondrial dysfunction in patients with COVID-19 can determine the different susceptibility of patients to infection, and therefore markers used to detect mitochondrial dysfunction (membrane depolarization, mitochondrial enzyme activity) are promising for personalized assessment of the risk of infection with SARS-CoV-2 and stratification of complications [103,105].

Some of the unanswered questions regarding the pathogenetic aspects of COVID-19 may be resolved by individual assessment of the immune response of patients to the virus, in particular, the asymptomatic clinical forms of infection and differences in the severity of the disease depending on age and sex [106,107,108].

In this case, individual profiling of the immune status with subsequent measurement of cytokine activity becomes a promising biomarker for making therapeutic decisions at the level of individual patient. It seems that we are on the verge of a multi-marker laboratory-based diagnosis of COVID-19, which, in addition to improving the effectiveness of therapy, will provide higher cost-effectiveness.

So, is it already the time personalized medicine to control the COVID-19 pandemic?

## 8. Conclusions

Some successes in the control of the COVID-19 pandemic have been achieved, inter alia, thanks to the well-proven basic laboratory diagnostic tools based on, primarily, molecular/genetic and immunoassay methods. It took decades to optimize these technologies, but they are now a key to identifying and controlling the spread of COVID-19. The experience gained in the elimination of the SARS and MERS epidemics served as a basis for the development of a laboratory strategy for the detection of the SARS-CoV-2 virus and antibodies to it during the COVID-19 pandemic. Their competent implementation and timely and correct interpretation of the results allow necessary organizational decisions to be made in time. However, there is still a need to find and develop rapid, reliable, cost-effective, and, simultaneously, sensitive and specific tools for diagnosing and controlling the spread of the SARS-CoV-2 infection.

Knowledge about SARS-CoV-2 is growing exponentially, and unexpected discoveries are also possible in the future. For example, the recently discovered strain SARS-CoV2 Spike D614G is characterized by a mutation in the main determinant, protein S [86]. Its increased virulence and rapid spread throughout the world largely confirm the need to develop rapid technologies targeted at various regions of the novel coronavirus genome.

Control over the COVID-19 pandemic requires the combined use of various types of laboratory-based diagnostic testing techniques, the sensitivity and specificity of which increase as SARS-CoV-2 is studied. The testing strategy should be designed in such a way as to conduct, depending on the timing of examination and severity of the infection, large-scale and repeated studies based on the principle: screening–monitoring–control. The search and development of new methods on new analytical platforms for laboratory-based rapid diagnosis of COVID-19 is still a highly important and urgent healthcare issue.

In the final part, the authors place special emphasis on the timeliness of using the concept of personalized medicine to combat the COVID-19 pandemic in connection with the study of the reasons for the variability of individual vulnerability to SARS-CoV-2, increasing the efficiency and cost-effectiveness of patient treatment.

## Figures and Tables

**Table 1:** Mean time and seroconversion rate in the case of COVID-19 as inferred from the results of examination of 173 patients with confirmed diagnosis by [56].

Classes of Antibodies against SARS-CoV-2	Estimated Level of Seroconversion	Mean Time of Seroconversion (Days)	Seroconversion within 15 Days after Symptom Onset
Total antibodies	93.1% (161/173)	11	100.0%
IgM	82.7% (143/173)	12	94.3%
IgG	64.7% (112/173)	15	79.8%

**Table 2:** Comparison of the methods of loop mediated isothermal amplification (LAMP) and classical polymerase chain reaction (PCR).

Criteria	LAMP	PCR
Temperature cycles	Isothermal amplification (60–65 °С)	Different temperature cycles required
Number of primers	4(6) specially designed primers	2 primers
Analysis time	Up to 45 min	Up to 6–8 h
DNA output	DNA yield—10–20 μg	DNA yield—up to 0.2 μg
Visual detection	Possible	Impossible
Economy and ease of use	Economical and easy to carry out	Requires expensive equipment and trained personnel
Sample inhibitor sensitivity	Insensitive	Sensitive
Multiplexing capability	Possible	Impossible
Knowledge of the method, clinical evaluation	Little known, clinical evaluation ongoing	Well known, clinically proven

**Table 3:** Examples of commercial LAMP test systems (RT-LAMP) approved for SARS-CoV-2 detection.

Country of Origin and Developer Company	Name	Description	Refs.
Melbourne University, Australia	N1-STOP-LAMP	Qualitative (RT-LAMP) detection of the CDC N1 region of the nucleocapsid (N) SARS-CoV-2 gene in oral and nasopharyngeal mucus samples, infection. Result <20 min.	[70]
Seasun Biomaterials Inc., South Korea	AQ-TOP™ COVID-19 Rapid Detection Kit.	Qualitative (RT-LAMP) detection of RNA in samples of mucus from oropharynx and nasopharynx, BAL fluid and sputum in the acute phase of infection. Result <30 min.	[71]
Color Genomics, Inc., USА	Color Genomics SARS-COV-2 RT-LAMP Diagnostic Assay	Qualitative (RT-LAMP) detection of RNA in samples of mucus from oropharynx and nasopharynx, bronchoalveolar lavage (BAL) fluid and sputum in the acute phase of infection. Result <30 min.	[72]

**Table 4:** Lateral flow immunoassay (LFIA) test systems approved for the diagnosis of COVID-19 [78].

Country of Origin and Developer Company	Sensitivity/Specificity of Test Systems (%)	Description	Used Biosubstrates, Analysis Time	Refs.
US/China, Cellex Inc.	93.8/95.6	Detection of IgM/IgG to the nucleocapsid SARS-CoV-2	Serum, plasma, whole blood (K2-EDTA, Na citrate), 20 min	[79]
US, ChemBio	92.7 (IgM) и 95.9 (IgG)/99.0 (IgM и IgG)	Detection of IgM/IgG to the nucleocapsid SARS-CoV-2	Serum, plasma, whole blood (K2-EDTA, heparin), 15 min	[80]
US, Autobio Diagnostics Co. Ltd. (+ Hardy Diagnostics)	95.7 (IgM) и 99.0 (IgG)/99.0 (IgM и IgG)	Detection of IgM/IgG к antigenic proteins SARS-CoV-2	Serum, plasma, whole blood (K2-EDTA, heparin), 15 min	[81]
US/China, Healgen Scientific LLC	96.7 (IgG), 86.7 (IgM), 96.7/98.0 (IgG), 99.0 (IgM), 97.0	Detection of IgM/IgG к antigenic proteins SARS-CoV-2	Serum, plasma, whole blood (K2-EDTA, heparin), 10 min	[82]
China, Hangzhou Biotest Biotech Co., Ltd.	92.5 (IgM), 91.56 (IgG)/98.1 (IgM), 99.52 (IgG)	Detection of IgM/IgG to S1 locus of S protein SARS-CoV-2	Serum, plasma, whole blood (K2-EDTA, heparin), 20 min	[83]
US/China, Aytu Biosciences/Orient Gene Biotech	87.9 (IgM) & 97.2 (IgG)/100.0 (IgG и IgM	Detection of IgM/IgG к antigenic proteins SARS-CoV-2	Serum, plasma, whole blood (K2-EDTA, Na citrate), 10 min	[84]

## Data Availability

Data sharing is not applicable to this article.
